# Structure of the endogenous insect acetyl-coA carboxylase carboxyltransferase domain

**DOI:** 10.1016/j.jbc.2024.107800

**Published:** 2024-09-19

**Authors:** Dong Wang, Fan Bu, Ge Yang, Hannah Brenke, Bin Liu

**Affiliations:** 1 The Hormel Institute, University of Minnesota, Austin, Minnesota, USA; 2 Department of Pharmacology, University of Minnesota Medical School, Minneapolis, Minnesota, USA; 3 Department of Biology, Gustavus Adolphus College, Saint Peter, Minnesota, USA

**Keywords:** *Trichoplusia ni*, carboxyltransferase domain, acetyl-coenzyme A carboxylase, cryo-EM, pest control

## Abstract

Acetyl-coenzyme A carboxylases (ACCs) are pivotal in fatty acid metabolism, converting acetyl-CoA to malonyl-CoA. While ACCs in humans, plants, and microbes have been extensively studied, insect ACCs, crucial for lipid biosynthesis and physiological processes, remain relatively unexplored. Unlike mammals, which have ACC1 and ACC2 in different tissues, insects possess a single ACC gene, underscoring its unique role in their metabolism. Noctuid moths, such as *Trichoplusia ni*, are major agricultural pests causing significant crop damage and economic loss. Their resistance to both biological and synthetic insecticides complicates pest control. Recent research has introduced cyclic ketoenols as novel insecticides targeting ACCs, yet structural information to guide their design is limited. Here, we present a 3.12 Å cryo-EM structure of the carboxyltransferase (CT) domain of *T. ni* ACC, offering the first detailed structural insights into insect ACCs. Our structural comparisons with ACC CT domains from other species and analyses of drug-binding sites can guide future drug modification and design. Notably, unique interactions between the CT and the central domain in *T. ni* ACC provide new directions for studying the ACC holoenzyme. These findings contribute valuable information for pest control and a basic biological understanding of lipid biosynthesis.

Acetyl-coenzyme A carboxylases (ACCs) are crucial for fatty acid metabolism, facilitating the conversion of acetyl-CoA to malonyl-CoA, an essential step in fatty acid synthesis (1). In eukaryotes, ACCs are large, multidomain enzymes consisting of biotin carboxylase (BC), biotin carboxyl carrier protein (BCCP), and carboxyltransferase (CT) domains (2). The BC domain initiates the reaction by carboxylating the biotin group attached to BCCP, while the CT domain transfers the activated carboxyl group to acetyl-CoA, completing the conversion to malonyl-CoA (1, 2). Eukaryotic ACCs, besides the canonical components, include two non-catalytic regions, the large central domain (CD) and the BC–CT (BT) interaction domain, with the functions of the CD domains remaining largely unknown (3).

ACCs represent promising targets for drug development (4) aimed at treating obesity-related diseases (5, 6, 7), tumor growth (3, 8), and microbial infections (9, 10, 11). They are also the target site for various commercial herbicides (12). For instance, CP-640186, an effective inhibitor of mammalian ACCs targeting the CT domain, has been shown to reduce body fat mass and improve insulin sensitivity, underscoring the potential of ACCs in anti-obesity and anti-diabetes therapies (6, 7). Moreover, herbicides such as haloxyfop (an aryloxyphenoxypropionic acid, or FOP) and sethoxydim (a cyclohexanedione, or DIM) have been used for over 50 years to target CT domain of ACCs, disrupting fatty acid biosynthesis in sensitive plants and causing their death (12, 13, 14).

While human and microbial ACCs have been extensively studied, insect ACCs have received relatively little attention. Mammals possess two ACC members: ACC1 in the cytosol of liver and adipose tissues and ACC2 associated with the mitochondrial outer membrane in the heart and muscle (15, 16), whereas insects and other invertebrates have only one ACC gene (17, 18). This single ACC gene is crucial for various physiological processes and lipid biosynthesis in insects, highlighting its importance in their metabolic activities.

The insecticide market has traditionally focused on compounds that target nerve and muscle functions, but recent developments have introduced new chemistries with alternative modes of action (19, 20). Notably, tetronic and tetramic acid derivatives, or cyclic ketoenols, have emerged as inhibitors of lipid biosynthesis by targeting acetyl-CoA carboxylase (20, 21, 22). These ketoenol insecticides are advantageous due to their favorable ecotoxicological profile, as they are compatible with managed bee pollinators and biological control agents (20, 23, 24).

Noctuid moths are significant agricultural pests, known as cutworms or armyworms, and damage a wide range of crops by feeding on various plant parts (25, 26). Their impact results in substantial economic losses. The cabbage looper (*Trichoplusia ni*), a polyphagous insect from the Noctuid family, is found globally and infests many vegetables in field and greenhouse settings (27, 28). Resistance to both biological and synthetic insecticides has exacerbated the challenge of managing this pest (29, 30, 31), prompting researchers to seek more effective control methods.

Understanding the structure and function of insect ACCs can provide insights into their unique metabolic pathways and inform the development of more selective and environmentally friendly insecticides. Recent studies have shown that spirotetramat-enol, a ketoenol insecticide, binds to the CT domain of ACC in insects and mites (20). Unlike plant ACCs, where specific mutations in the CT domain can disrupt herbicidal binding, similar mutations in spider mite ACC did not affect spirotetramat-enol inhibition (20). This suggests different binding mechanisms for ketoenols and herbicidal ACC inhibitors and highlights structural differences between plant and insect ACCs.

The current lack of structural knowledge of insect ACC CT domains complicates the design of targeted pesticides. To address this gap, our group has reported a 3.12 Å cryo-EM structure of the carboxyltransferase domain of *Trichoplusia ni* acetyl-Coenzyme A carboxylase. This study provides the first detailed structural insights into the insect ACC CT domain and offers valuable comparisons with ACC CT domains from other species. Additionally, we performed extensive structural comparison analyses on residues surrounding drug-binding sites. Moreover, our density map reveals unique organizational features between the CT and CD domains in *Trichoplusia ni* ACC. These findings provide crucial insights for future pesticide design and expand our understanding of ACCs.

## Results and discussion

### The overall structure of carboxyltransferase domain of *T. ni* ACC

**Figure 1:**
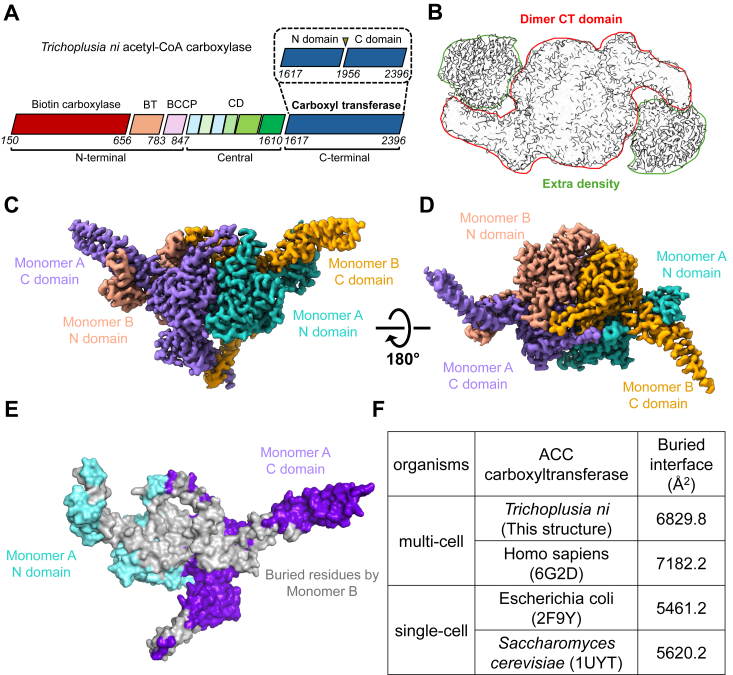
**The overall structure of the *T. ni* acetyl-coA carboxylase.***A*, schematic diagrams of the architecture of *T. ni* ACC, with an inset showing a zoom-in of the carboxyltransferase domain. The *T. ni* ACC comprises five domains. *B*, the cryo-EM map reveals the density of the ACC dimer CT domain along with the extra density, which might be the CD domains. Parameters: Step 1, contour level set to 0.125 in ChimeraX. *C* and *D*, the cryo-EM density map of the *T. ni* ACC carboxyltransferase domain, shown from the front and top views. N domain in monomer A, *light sea green*; C domain in monomer A, *medium purple*; N domain in monomer B, *light pink*; C domain in monomer B, *orange*. *E*, *T. ni* ACC carboxyltransferase monomer A in surface mode, with residues buried by monomer B highlighted in *grey*, mapped onto its structure. *F*, the buried interface of the ACC carboxyltransferase dimer from different species. The buried interface was calculated using the online server PDBePISA.

To study the structure of *T. ni* ACC, we first isolated the endogenous ACC protein directly from the functional *T. ni* cell line using Strep-Tactin XT resin, an engineered streptavidin agarose resin. This resin specifically recognizes biotin that is covalently linked to the BCCP domains of biotin-dependent carboxylases, including ACC. This allowed us to extract ACC without the need for any additional affinity tags. Next, the extracted biotin-dependent carboxylases were further purified by a size exclusion column (Fig. S1*A*). SDS-PAGE analysis revealed that the 250 kDa *T ni* ACC was purified successfully (Fig. S1*B*), matching the common size of eukaryotic ACC, which contains approximately 2500 amino acids. Additionally, two other biotin-dependent carboxylases, methylcrotonyl-CoA carboxylase alpha (MCCA, ∼78 kDa) and beta (MCCB, ∼60 kDa), may also have been isolated (32). Subsequently, the morphology of the purified ACC was examined by negative staining TEM, revealing single particles in a homogeneous form with an approximate diameter of 200 Å (Fig. S1*C*). Therefore, samples were collected for cryo-EM, resulting in a final 3.12 Å cryo-EM density map (Figs.1, S2, S3, and Table S1).

The complete gene of *T. ni* ACC contains three canonical domains: biotin carboxylase, biotin carboxyl carrier protein, and carboxyltransferase. Additionally, it has two domains, the CD and the BC–CT interaction domain, that are present only in eukaryotes (Fig. 1*A*). However, the 3.12 Å resolution cryo-EM density map revealed only the CT domain in high resolution and the CD domain in low resolution (Fig. 1*B*), with the other domains being invisible, likely due to the intrinsic dynamics and metastability of these domains in *T. ni* ACC. Since *T. ni* ACC is directly isolated from the cell line, it represents the most native and functional form.

The solved ACC CT domain is full length, spanning from amino acid 1622 to 2396, with approximately 175 Å in diameter (Fig. 1*C*). CT forms a head-to-tail dimer, where the N domain of one monomer interacts with the C domain of the other (Fig. 1, *D* and *E*). Both the N and C domains extensively interact with each other and with the ones from different protomers mainly by hydrophobic interactions and salt bridges. Additionally, when comparing the buried interface area among different species, single-cell organisms tend to have a lower buried interface, while human ACC CT exhibits the highest buried interface, with *T. ni* ACC CT nearly as extensive as the human ACC CT (Fig. 1*F*). This is likely because the CT domain of multi-cell organism ACCs contains a small subdomain that enhances dimer stability, which is not present in the CT domains of single-cell organism ACCs. Furthermore, for the kinetics of purified recombinant ACCs from various species, the Km values for malonyl-CoA in *E. coli* and yeast CTs have been reported as 100 μM (33) and 75 μM (13), respectively, while the Km for acetyl-CoA in human ACC1 is 34 μM (34). Despite differences in the substrates used for enzyme activity assays, these findings may suggest a potential positive correlation between the Km of ACC and the dimer interface area. The observed correlation indicates that a larger interface in multi-organism ACCs, such as those in humans and *T. ni*, enhances dimer stability, potentially lowering the Km and increasing substrate affinity.

### Superimposition of ACC CT domain in complex with CoA and herbicides

**Figure 2:**
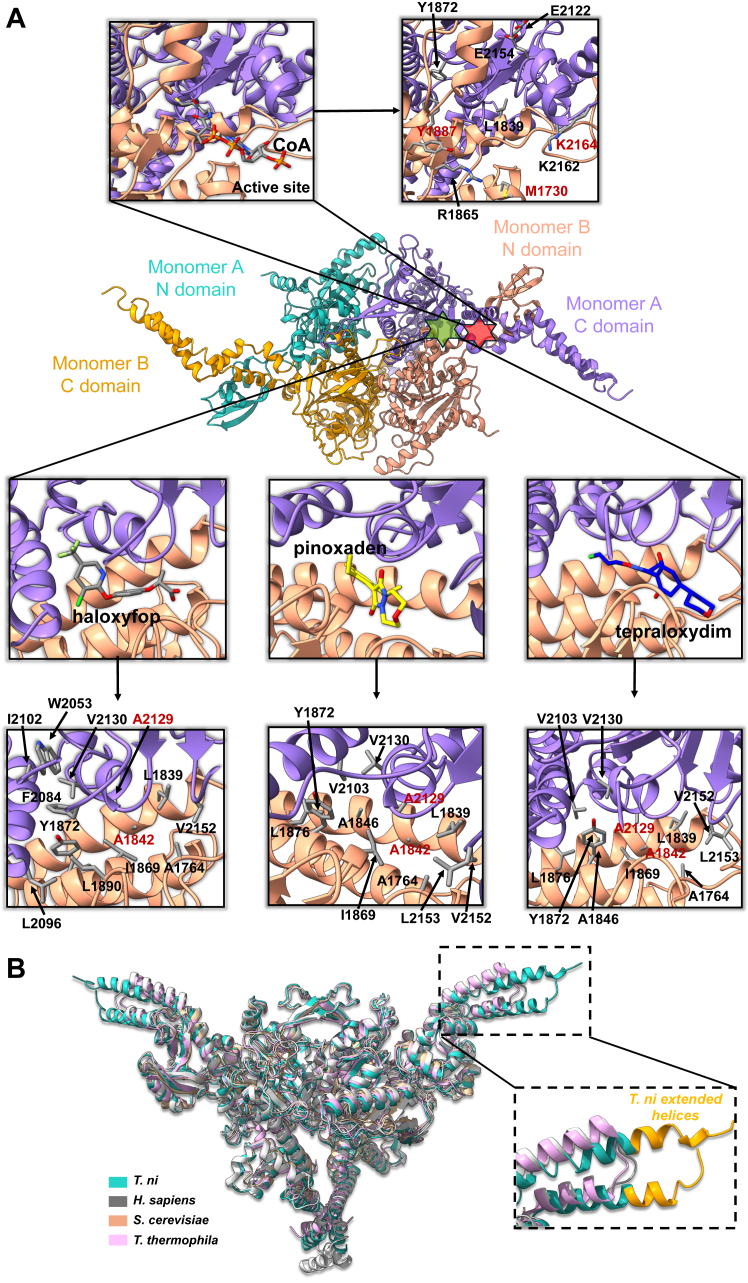
**Superimpositions of ACC CTs.***A*, *T. ni* ACC CT is superimposed with *T. thermophila* ACC CT in complex with herbicides: haloxyfop (1UYS), pinoxaden (3PGQ), and tepraloxydim (3K8X), as well as *S. cerevisiae* ACC CT in complex with CoA substrate (1OD2). All color codes match Figure 1. The upper zoom-in ones show the CoA docked at the active site of *T. ni* ACC CT domain and its relevant residues. All relevant residues are represented in stick representation and highlighted in *gray*. The down zoom-in ones display the dockings of herbicides on *T. ni* ACC CT domain and their relevant residues. Residues labeled in *red* represent differences from *T. thermophila* and *S. cerevisiae* ACCs, while the *black* ones represent the conserved residues. *B*, *T. ni* ACC CT (*light sea green*) is superimposed with *S. cerevisiae* ACC CT (1UYT, wheat), *T. thermophila* ACC CT (5I6H, *light pink*), and *H. sapiens* ACC CT (6G2H, *grey*). The extended helices present only in *T. ni ACC* CT are shown in *orange*.

The CT active site is located at the dimer interface (Fig. 2*A*, upper), necessitating dimerization for catalytic activity. This highlights the importance of the buried interface in ensuring stable substrate binding within a complex cellular environment. The crotonase fold, known for recognizing CoA esters, enables the N domain in CT to bind the acyl-CoA substrate. The prevalence of the CT component and its conserved CoA binding pocket in all ACCs highlights the crucial catalytic function of CT. Consequently, various drug candidates and pesticides are designed to target the CT domain. Previous research has shown the binding pockets of herbicides (haloxyfop, pinoxaden, and tepraloxydim) on CT (13, 35, 36). Those binding pockets are close to the CoA catalytic site but are located deeper within the hydrophobic interfaces in the dimer, whereas the conserved CoA catalytic site contains more charged residues (Fig. 2*A*, down). We superimposed their structures onto our unbound CT structure and found that the interacting residues are largely conserved in *T. ni* ACC CT, with a few mutations that did not significantly change the pocket polarity. This conservation represents their ability to across-react to insects to some extent. For instance, haloxyfop, pinoxaden, and tepraloxydim binding pockets (Fig. 2) exhibited only two residue differences: valine 2129 mutated to alanine and serine 1842 to alanine. The *T. ni* ACC CT binding pocket appears to retain a hydrophobic environment the same as *S. cerevisiae* ACC CT, suggesting that both structures might exhibit comparable coA-binding affinities. This similarity could imply a conserved functional role across these species. Aligning with what has been reported in functional studies (20), haloxyfop, pinoxaden, and tepraloxydim are effective against some pests. Although the structures of CT-herbicide complexes are extensively available, little information has been shown on the new generation of pesticides. Several mutations in the herbicide binding pockets did not affect spirotetramat-enol inhibition of insect ACC while functional studies indicate it binds to CT (20). We suggest that there might be an undiscovered binding pocket on CT or that a conformational change is required. Extensive mutational studies and further structural analysis of the complex are likely to resolve this question.

### Structural comparison of the ACC CT domains among eukaryotic ACCs

Structures of prokaryotic and eukaryotic ACC domains, holoenzymes, and filaments have been a hot topic over the decades, with a particular focus on humans and fungi (Table S2). To better understand how insect (*T. ni*) ACC differs from these known structures, we performed sequence alignment and structural superimposition on these representative structures (Figs. 2*B* and S4). Our results show that the majority of the structure is largely conserved; however, the C terminus of the C domain (residues 2180–2230) in *T. ni* has elongated alpha helices (Figs. 2*B* and S4). The extended α19 and α20 helices likely impact the dynamics of the *T. ni* ACC holoenzyme function and the pathway for lipid biosynthesis, although further functional studies are needed to confirm the structural observation.

**Figure 3:**
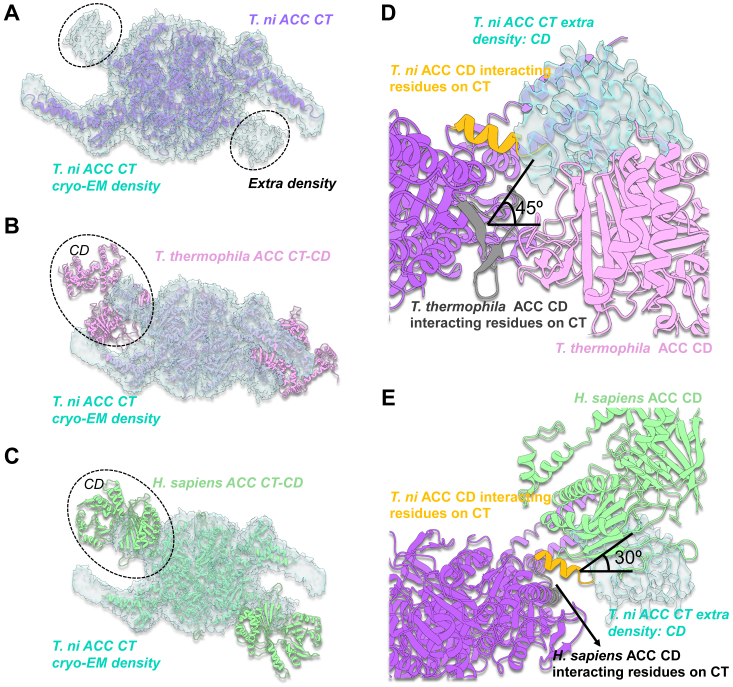
**Superimposition with *T. thermophila* and *H. sapiens* ACC CT-CDs.***A*, the extra density identified in the cryo-EM map is close to the N terminus of the N domain. *B*, the *T. thermophila* ACC CT-CD structure superimposed onto the *T. ni* ACC CT cryo-EM map shows that the CD is in the same spatial orientation as the extra density. *C*, the *H. sapiens* ACC CT-CD structure superimposed onto the *T. ni* ACC CT cryo-EM map. *D*, *T. thermophila* ACC CD and *T. ni* ACC CT structures are superimposed onto the *T. ni* ACC CT cryo-EM density map, with the extra density likely originating from *T. ni* ACC CD, which interacts with CT at a different angle (∼45 degrees) than *T. thermophila* ACC CD. *E*, *H. sapiens* ACC CD and *T. ni* ACC CT structures are superimposed onto the *T. ni* ACC CT cryo-EM density map, with the extra density likely *T. ni* ACC CD, which interacts with CT at a different angle (∼30 degrees) than *H. sapiens* ACC CD.

Furthermore, when assigning residues to the cryo-EM density, we found an extra density adjacent to the N terminus of the N domain, although the resolution is low due to flexibility. We superimposed the crystallographic structure of the *T. thermophila* ACC CT-CD domain and the cryo-EM structure of the *H. sapiens* ACC CT-CD structure onto the *T. ni* ACC CT cryo-EM density map, confirming that the extra density is likely associated with the CD domain (Fig. 3, *A*–*C*). Additionally, we found that the CD domain of *T. ni* interacts with CT at a different angle, deviating approximately 45° counterclockwise from the angle in *T. thermophila* and around 30° clockwise from the angle in *H. sapiens* (Fig. 3, *D* and *E*). This difference results in *T. ni* interacting with different residues on the CT than *T. thermophila* and *H. sapiens*, with the former exclusively interacting with the C terminus α helix. The variability in the connections of CD to CT has been observed previously; however, the limitations of crystallography could lock CD-CT in a certain conformation by crystal packing (10). The native ACC likely represents the relative location of CD-CT. In addition, we used AlphaFold (37) to predict the CD domain and modeled it with the determined CT domain of ACC (Fig. S5). The predicted CD domain contains N-terminal and C-terminal subdomains, which are notably conserved compared to the resolved CD domains from other species (Fig. S6). However, additional helices and loops (residues 371–404, 426-464) were found in the C-terminal region of the CD domain (Figs. S5 and S6), indicating a distinct feature of insect ACC.

Whether these differences are insect-specific needs further investigation by obtaining the holoenzyme structure through cryo-EM, especially to uncover the insect holoenzyme in its phosphorylated inhibited or non-phosphorylated activated state. Our study provides evidence that there might be distinct differences in the motional dynamics of *T. ni* ACC compared to that of other species. These differences could affect the enzyme's catalytic efficiency or regulatory mechanisms, potentially leading to species-specific variations in metabolic processes.

### Concluding remarks

This study presents the first detailed structural analysis of the carboxyltransferase domain of *T. ni* acetyl-coA carboxylase. Using cryo-EM on ACC isolated from a *T. ni* cell line, we obtained a 3.12 Å density map revealing the CT details. While the CT domain structure is largely conserved, *T. ni* exhibits unique elongated alpha helices at the C terminus, potentially affecting the holoenzyme's function and lipid biosynthesis. Comparison with other ACCs shows that the buried interface area of *T. ni* ACC CT is almost as extensive as that in human ACC CT, indicating significant dimer stability for carrying function.

The CT domain of ACC serves as the binding site for three distinct classes of herbicides, including haloxyfop, tepraloxydim, and pinoxaden. Crystal structures of the yeast ACC CT domain complexed with these herbicides are now available (13, 35, 36). Studies have shown that haloxyfop binds near the active site of the CT domain, at the dimer interface, and requires a significant conformational change in this interface for effective binding (13). In contrast, tepraloxydim interacts with a different region of the dimer interface, requiring only small but critical conformational adjustments (36). Pinoxaden exhibits a binding mode similar to tepraloxydim, with small conformational changes needed at the dimer interface (35). These binding characteristics elucidate the structure-activity relationships of these inhibitors and provide a molecular basis for their differential sensitivity to resistance mutations. In our study, structural superimposition of CT domains in complex with herbicides demonstrated that *T. ni* ACC CT maintains conserved interacting residues, with slight modifications that may enhance its binding affinity, providing valuable insights for designing selective pesticides.

Furthermore, our findings indicate the presence of extra density adjacent to the N terminus of the N domain, likely associated with the CD domain. The CD domain in *T. ni* interacts with the CT domain at a different angle compared to *T. thermophila* and *H. sapiens*, deviating by approximately 30°-45°. This unique interaction angle suggests that *T. ni* ACC CD interacts exclusively with the C terminus α helix of the CT domain, highlighting a potential insect-specific feature that warrants further investigation.

Therefore, our study sheds light on the architectural dynamics of *T. ni* ACC and provides a foundation for future research to explore the functional implications of these structural differences, particularly in the context of holoenzyme activity in different phosphorylation states. These findings contribute to a deeper understanding of ACC structure and function, with implications for pest control strategies and biochemical studies of lipid metabolism.

## Materials and methods

### Cell culture

*Trichoplusia ni* cells (Expression Systems) were cultured in suspension in ESF921 medium (Expression Systems) at 27 °C, 120 rpm in an incubator shaker (Benchmark). The cells were cultured until they reached a density of 4.0 × 10⁶ cells per mL, then harvested by centrifugation at 4000 rpm for 10 min. The cell pellets were flash-frozen in liquid nitrogen and stored at −80 °C.

### Endogenous protein purification

The frozen cells were thawed and resuspended in lysis buffer (25 mM Hepes pH 8.0, 150 mM NaCl, 1 mM tris(2-carboxyethyl) phosphine (TCEP), 0.5% NP-40) supplemented with Xpert Protease Inhibitor Cocktail (GenDEPOT). The resuspended cells were sonicated and then centrifuged at 29,000 rpm for 1 h at 4 °C. The resulting supernatant containing the target proteins was filtered through a 0.22-μm filter and then incubated with Strep-Tactin XT resin (IBA Lifesciences) for 30 min at 4 °C and washed four times with wash buffer (25 mM Hepes pH 8.0, 150 mM NaCl, 1 mM TCEP). The bound protein was eluted with elution buffer (25 mM Hepes pH 8.0, 150 mM NaCl, 1 mM TCEP, and 50 mM biotin).

The eluted protein was further purified using a size-exclusion chromatography system (Superose 6 Increase 10/300 Gl, GE Healthcare) equilibrated with size-exclusion buffer (25 mM Hepes pH 8.0, 150 mM NaCl, 1 mM TCEP). The fractions near the maximum height of the peak were analyzed by sodium dodecyl sulphate–polyacrylamide gel electrophoresis (SDS-PAGE). Fractions containing ACC were combined and concentrated to 1.5 mg/ml. The final sample was flash-frozen, and stored at −80 °C.

### Negative staining EM

Negative staining EM was performed to examine the *T. ni* ACC (Fig. S1*C*). 4 μl sample (∼0.3 μM) from the gel filtration column was applied onto freshly glow-discharged 200-mesh carbon-coated copper grids (EM Sciences), incubated for 1 min with following blotting of liquid excess. Grids were washed with water 3 times and then stained with 0.75% uranyl formate solution for 30 s. The specimen was then finally gently blotted from the side with filter paper to remove excess liquid and air-dried before imaging. Imaging was performed using Biotwin Tecnai Spirit 120 kV electron microscope (ThermoFisher Scientific) with a Gatan 4K × 4K CCD camera with a defocus of −2 μm at a nominal magnification of 490,00× and 980,00×.

### Cryo-EM sample preparation and data collection

Four μl *T ni* ACC (∼2 μM) was applied to freshly glow-discharged Quantifoil R1.2/1.3300-mesh copper grids (EM Sciences) and blotted for 4 s at 4 °C under 100% chamber humidity and plunge-frozen in liquid ethane using a Vitrobot Mark IV (FEI). Images were collected using the K3 Summit detector (Gatan) in CDS mode with a BioQuantum GIF energy filter (slit width of 20 eV) at the Hormel Institute, University of Minnesota. The data collection was performed using the EPU software (Thermo Fisher Scientific) with a pixel size of 0.664 Å (nominal magnification of 1,300,00×) and a nominal defocus value between −1.0 to −2.0 μm. Each image consists of 40 dose-framed fractions and was recorded with a total dose of 53.7 e^−^/Å^2^. Cryo-EM data collection statistics are summarized in Table S1.

### Image processing

Cryo-EM data were processed using cryoSPARC v4.5.1 (38), and the detailed procedure is outlined in Fig. S2. Briefly, a total of 6463 dose-fractionated movies were subjected to Patch motion correction using MotionCor2 (39) and Patch CTF estimation using CTFFIND-4.1.13 (40), with data downsampled by three-fourths (0.885333 Å/pixel after downsampling). Images with the defocus values outside of −0.6 to −3.0 μm or CTF fit resolutions worse than 6 Å were excluded from further steps. A total of 3,236,084 particles were then picked using both the Blob picker and then Template picker in cryoSPARC v4.5.1 and subjected to the Remove Duplicate Particles Tool. Junk particles were removed through two rounds of 2D classifications. A total of 513,450 particles from the good 2D classes were used for Ab-initio reconstruction of four maps. The heterogeneous refinement produced 154, 035 particles from the good class, which was then subjected to further non-uniform and CTF refinements with C2 symmetry to generate a final map of 3.12 Å resolution. Map resolution was determined by gold-standard Fourier shell correlation (FSC) at 0.143 between the two half-maps. Local resolution variation was estimated from the two half-maps in cryoSPARC v4.5.1.

### Model building and refinement

The *T. ni* ACC model was first automatically built using ModelAngelo (41) and then further manually rebuilt in COOT (42). The cryo-EM densities were sufficient to define the region of residues 1622 to 2396 (CT domain). The N-terminal part is disordered due to high flexibility. The CD domain is visible at a lower contour but not built. Real-space refinements in Phenix (43) were performed to obtain the final model. In the real-space refinement, minimization global, local grid search, and adp were performed with the secondary structure, rotamer, and Ramachandran restraints applied throughout the entire refinement. The stereochemistry of all structural models was evaluated using MolProbity (44) in Phenix. Model and map statistics are summarized in Table S1. Figures were generated using UCSF Chimera X (45).

### Alignment and contact surface analysis

Sequence alignments of various *T. ni* ACC CT domains were performed using online tools, Clustal Omega (46) (https://www.ebi.ac.uk/jdispatcher/msa/clustalo) and ESPript 3.0 (47) (https://espript.ibcp.fr/ESPript/ESPript/index.php). Conserved residues are colored white on a red background. Highly similar residues are colored in red and framed in blue. Secondary structure information is labeled on the top. The buried interfaces of *T. ni* ACC CT complex were analyzed using PISA (48) at European Bioinformatics Institute (http://www.ebi.ac.uk/pdbe/prot_int/pistart.html).

## Data availability

The atomic coordinate of *T.ni* ACC CT has been deposited in PDB with accession number 9CV6. The cryo-EM density map has been deposited in the Electron Microscopy Data Bank with accession number EMD-45956.

## Supporting information

This article contains supporting information.

## Conflict of interests

The authors declare that they have no conflicts of interest with the contents of this article.
