# Supplementary material for: Structure of the endogenous insect acetyl-coA carboxylase carboxyltransferase domain
Source: J Biol Chem. 2024 Sep 19;300(10):107800. doi: 10.1016/j.jbc.2024.107800 (PMC11735997; doi:10.1016/j.jbc.2024.107800)
Supplement: Supplementary information [file mmc1.pdf]

Supplementary Information

Structure of the endogenous insect acetyl-coA carboxylase carboxyltransferase domain

Dong Wang[1]†, Fan Bu[1,2]†, Ge Yang[1], Hannah Brenke[1,3], Bin Liu[1]*

[1]The Hormel Institute, University of Minnesota, Austin, MN, USA

[2]Department of Pharmacology, University of Minnesota Medical School, Minneapolis, Minnesota, USA

[3]Gustavus Adolphus College, Saint Peter, Minnesota, USA

†These authors contributed equally to this work.

*Correspondence: liu00794@umn.edu (B.L.)

Fig. S1-S6

Table S1-S2

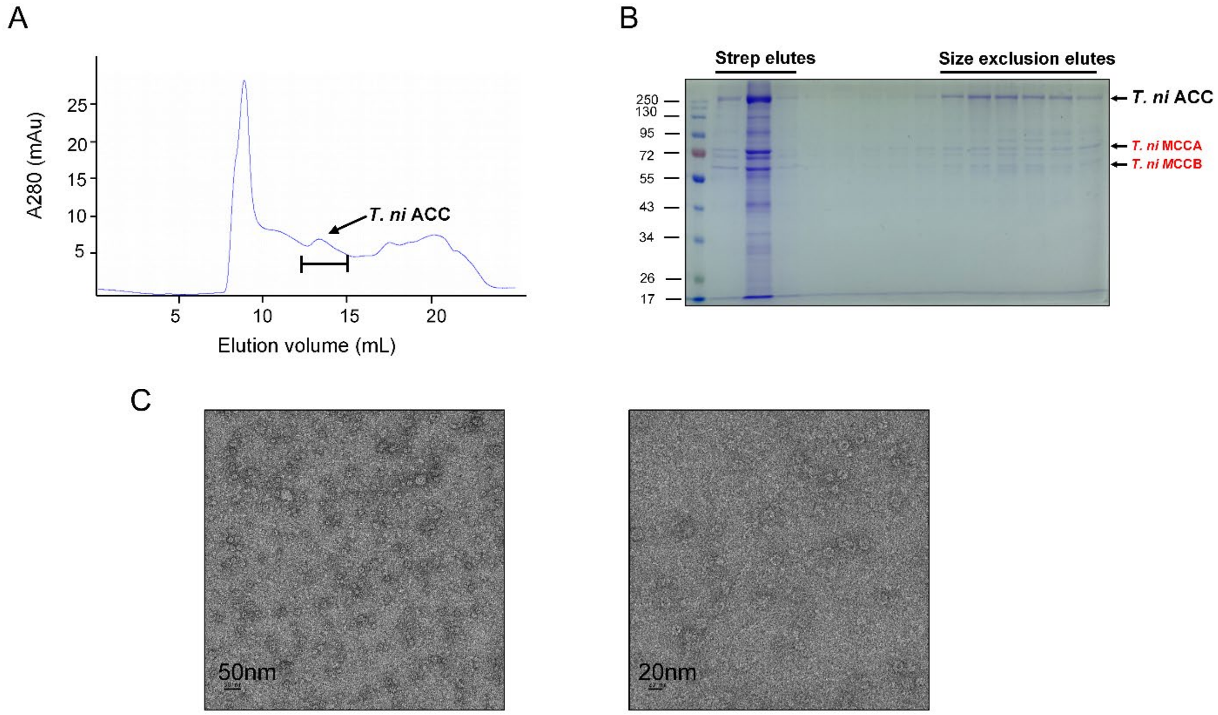

Fig. S1. The purified endogenous *Trichoplusia ni* acetyl-coA carboxylase. A-B. Size-exclusion chromatography and SDS-PAGE. Molecular weights of ladder markers (in kilodaltons, kDa) are shown on the left, and purified CT bands are labeled on the right. **C.** Negative staining micrographs of the *T. ni* ACC at 49,000× (left) and 98,000× magnifications (right), with scale bars indicated.

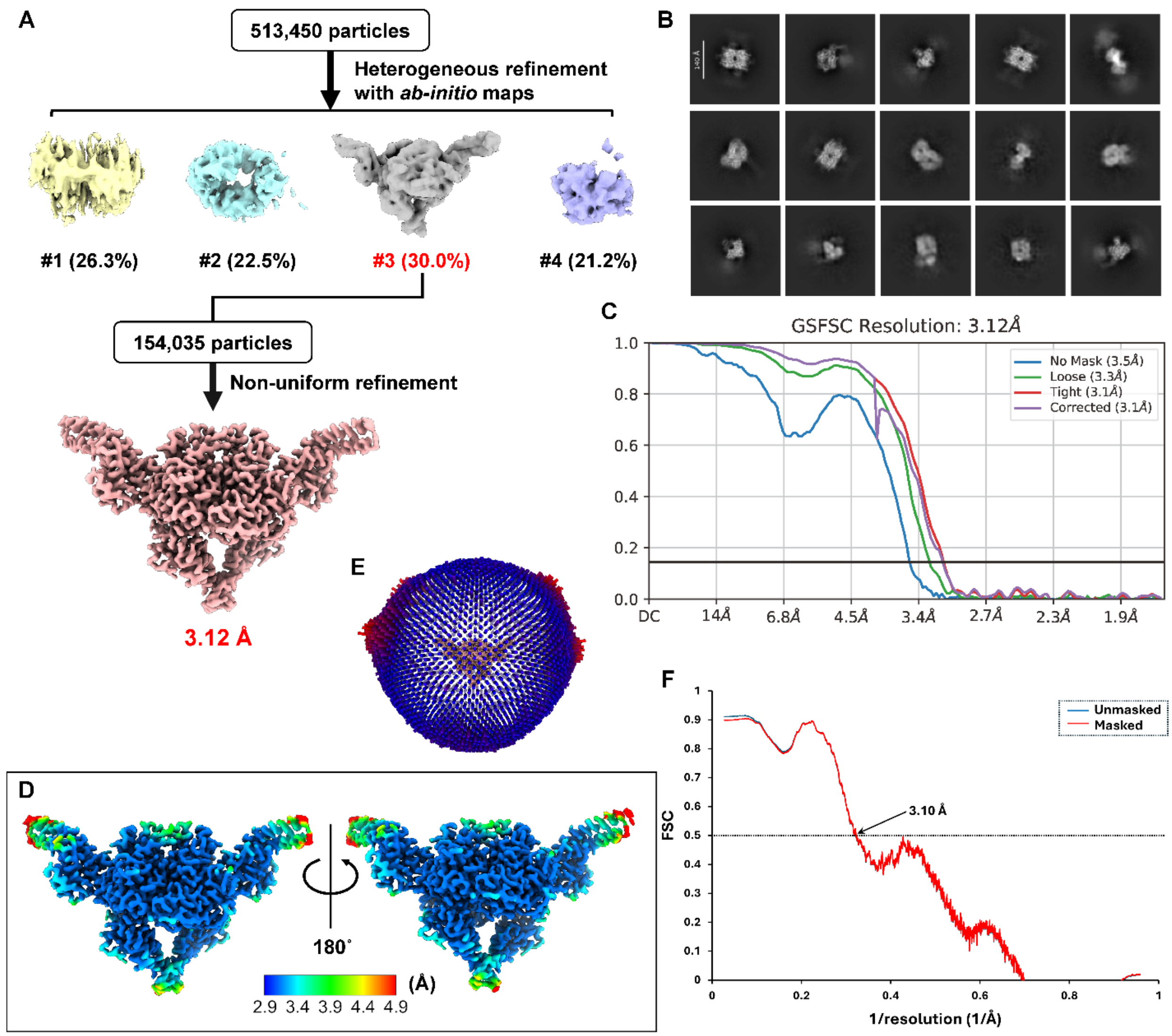

Fig. S2. Cryo-EM data processing workflow. A. Flow chart of cryo-EM data processing and map reconstruction for *T. ni* ACC. **B.** Representative 2D classes of *T. ni* ACC from the final classification. **C.** Gold-standard Fourier Shell Correlation (FSC) curves of the final map. **D.** The overall local resolution maps of the final 3D reconstruction. **E.** The angular distribution plot of the map. **F.** FSC curves of model-to-map validated for the final 3D reconstruction.

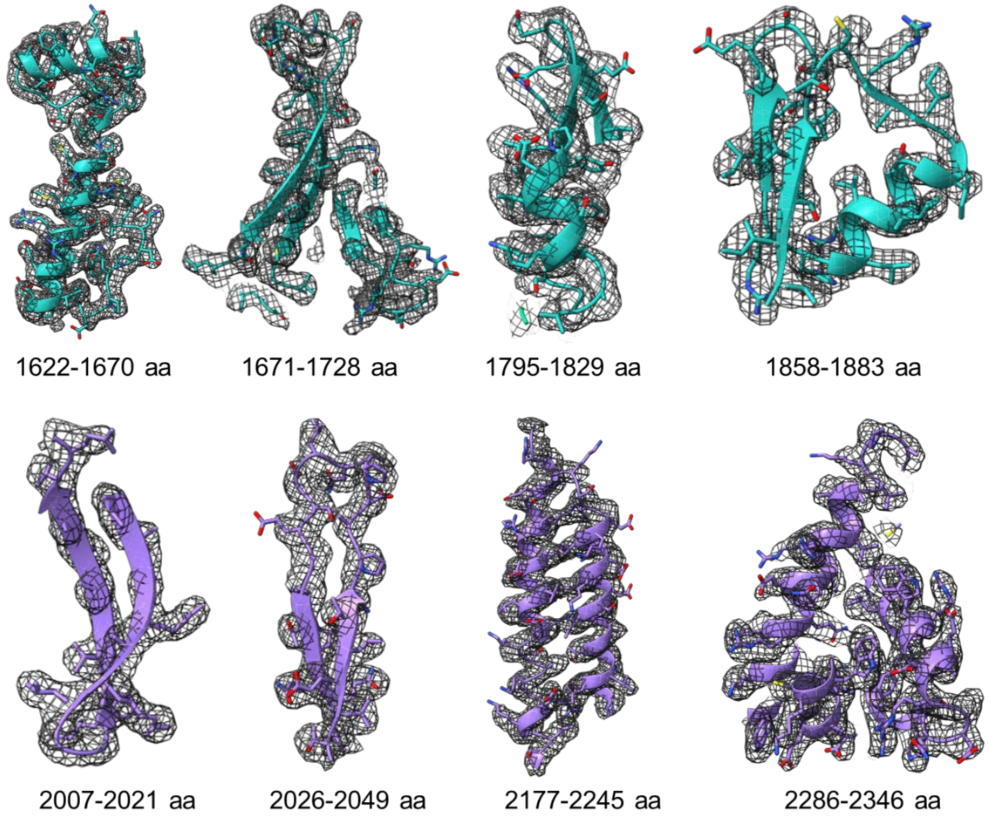

Fig. S3. Validation of the cryo-EM structural model. Cryo-EM densities are overlaid on the atomic model for representative residues of the CT domain in monomer A of *T. ni* ACC. Residues are depicted as sticks and colored according to the domain color in Figure 1.

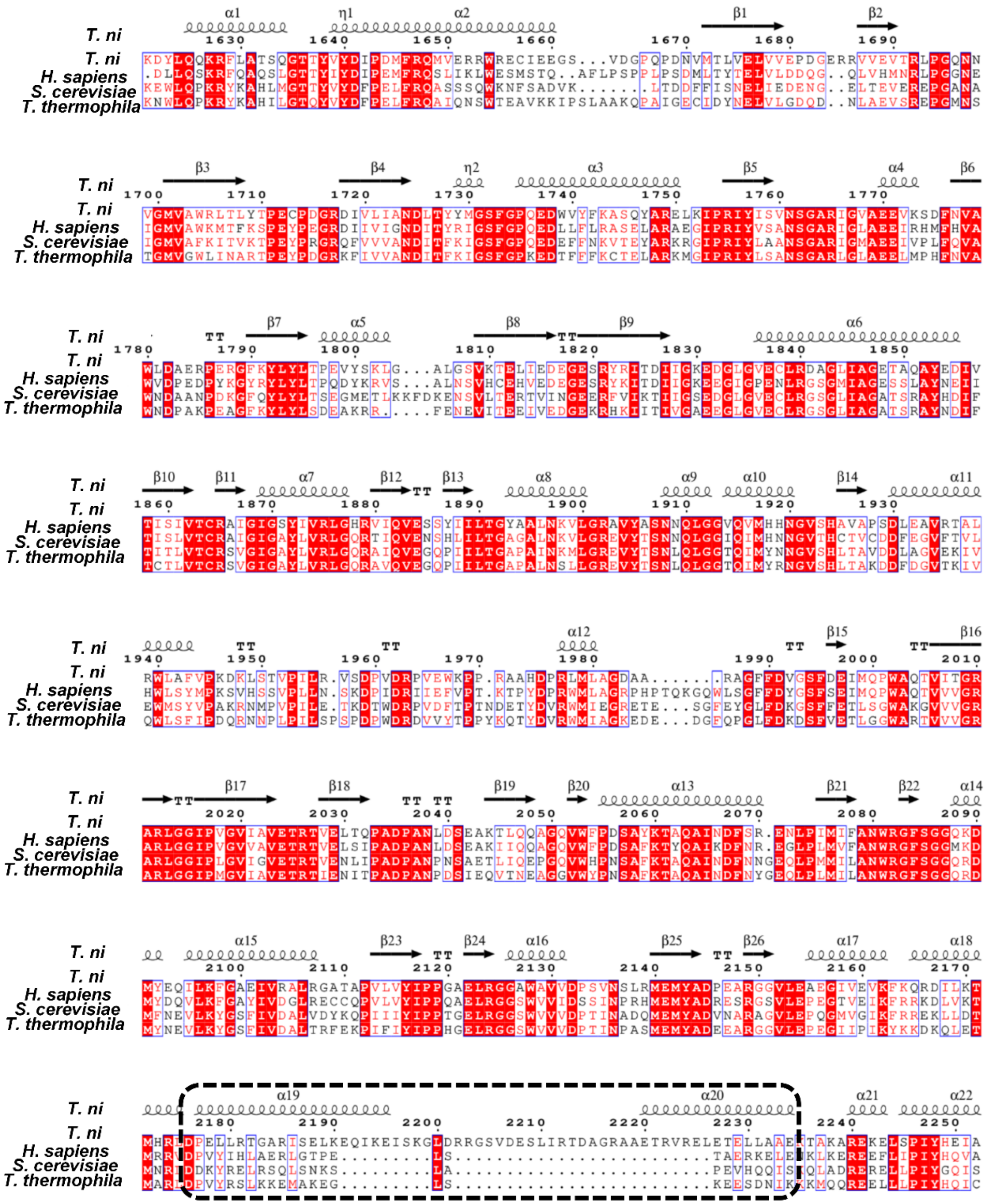

Fig. S4. Sequence alignment of the ACC CT domain in eukaryotes. Sequence alignment of the ACC CT domain was performed for *T. ni* (this structure), *S. cerevisiae* (1UYT), *T. thermophila* (5I6H), and *H. sapiens* (6G2H).

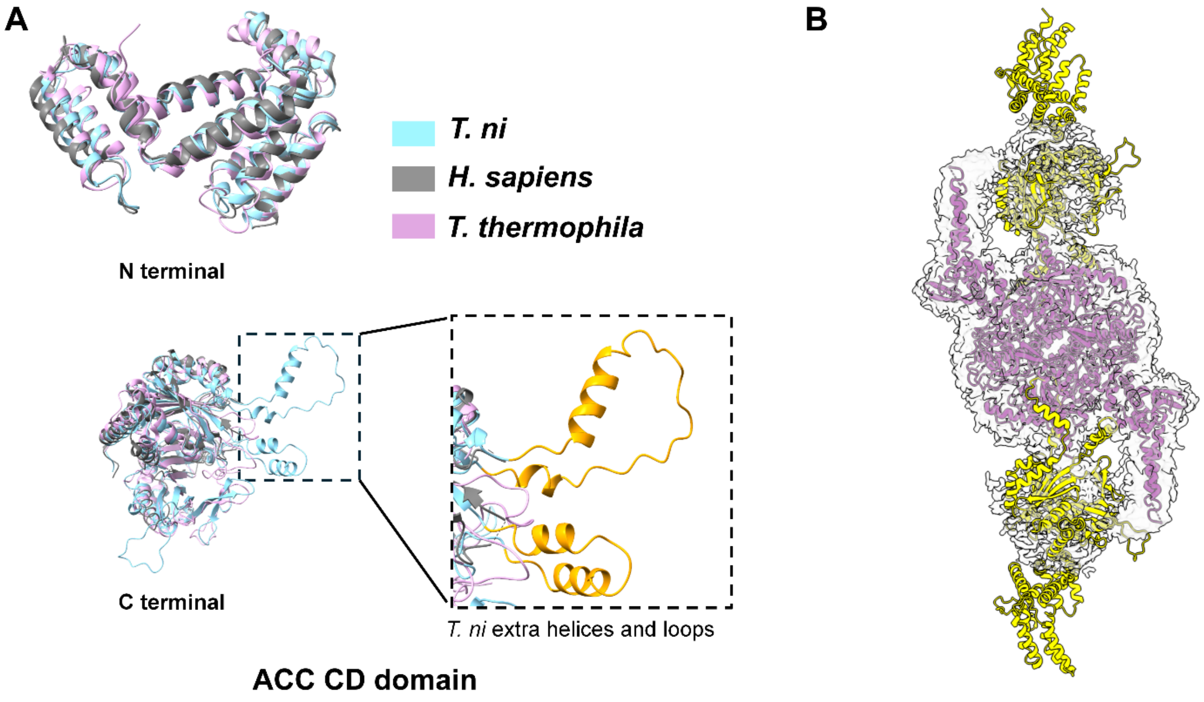

Fig. S5. The predicted CD domain structure of *T. ni* ACC by AlphaFold. A. Superimposition of the predicted N-terminal and C-terminal regions of *T. ni* ACC with the structures from *T. thermophila* (5I6H) and *H. sapiens* (6G2D) shows good alignment, except for some extra helices and loops found in the C-terminal region of the *T. ni* ACC CD domain. The predicted structure is colored blue, while the structures of *T. thermophila* and *H. sapiens* are pink and grey, respectively. The extra helices and loops are highlighted in yellow. **B.** The modeled structure of the *T. ni* ACC includes the determined dimer CT domain (purple) and the predicted CD domain (yellow).

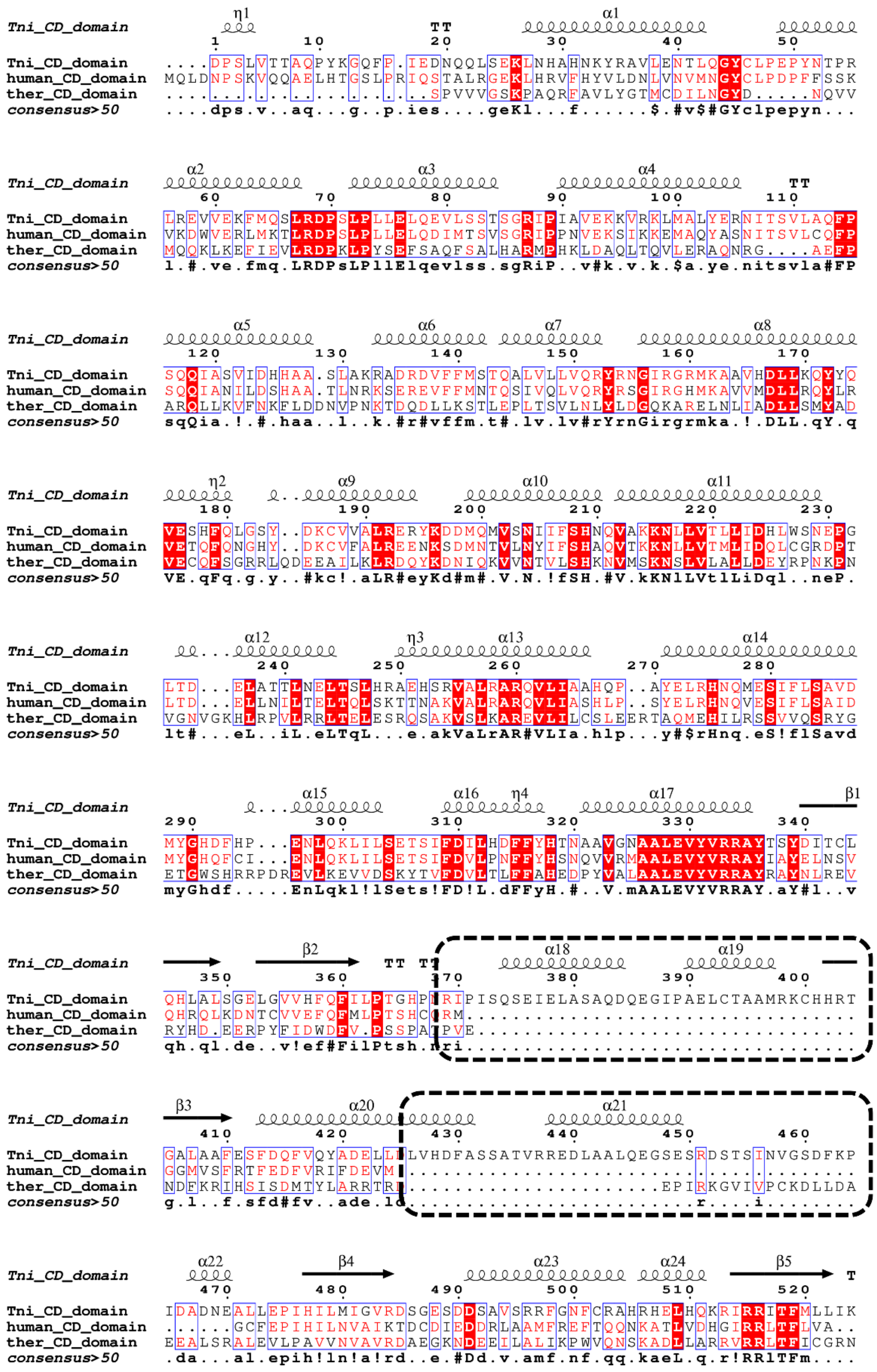

Tni_CD_domain ....DPSLVTTAQPYKGQFP.IEDNQQLSEKLNHAHNKYRAVLENTLQGYCLPEPYNTPR
human_CD_domain MQLDNPSKVQQAELHTGSLPRIQSTALRGEKLHRVFHYVLDNLVNVMNGYCLPDPFFSSK
ther_CD_domain .......................SPVVVGSKPAQRFAVLYGTMCDILNGYD.....NQVV
consensus>50 ....dps.v..aq...g..p.ies....geKl...f......$.#v$#GYclpepyn...
Tni_CD_domain LREVVEKFMQSLRDPSLPLLELQEVLSSTSGRIPIAVEKKVRKLMALYERNITSVLAQFP
human_CD_domain VKDWVERLMKTLRDPSLPLLELQDIMTSVSGRIPPNVEKSIKKEMAQYASNITSVLCQFP
ther_CD_domain MQQKLKEFIEVLRDPKLPYSEFSAQFSALHARMPHKLDAQLTQVLERAQNRG....AEFP
consensus>50 l.#.ve.fmq.LRDPsLPllElqevlss.sgRiP..v#k.v.k.$a.ye.nitsvla#FP
Tni_CD_domain SQQIASVIDHHAA.SLAKRADRDVFFMSTQALVLLVQRYRNGIRGRMKAAVHDLLKQYYQ
human_CD_domain SQQIANILDSHAA.TLNRKSEREVFFMNTQSIVQLVQRYRSGIRGHMKAVVMDLLRQYLR
ther_CD_domain ARQLLKVFNKFLDDNVPNKTDQDLLKSTLEPLTSVLNLYLDGQKARELNLIADLLSMYAD
consensus>50 sqQia.!.#.haa..l..k.#r#vffm.t#.lv.lv#rYrnGirgrmka.!.DLL.qY.q
Tni_CD_domain VESHFQLGSY..DKCVVALRERYKDDMQMVSNIIFSHNQVAKKNLLVTLLIDHLWSNEPG
human_CD_domain VETQFQNGHY..DKCVFALREENKSDMNTVLNYIFSHAQVTKKNLLVTMLIDQLCGRDPT
ther_CD_domain VECQFSGRRLQDEEAILKLRDQYKDNIQKVVNTVLSHKNVMSKNSLVLALLDEYRPNKPN
consensus>50 VE.qFq.g.y..#kc!.aLR#eyKd#m#.V.N.!fSH.#V.kKNlLVtlLiDql..neP.
Tni_CD_domain LTD...ELATTLNELTSLHRAEHSRVALRARQVLIAAHQP..AYELRHNQMESIFLSAVD
human_CD_domain LTD...ELLNILTELTQLSKTTNAKVALRARQVLIASHLP..SYELRHNQVESIFLSAID
ther_CD_domain VGNVGKHLRPVLRRLTELESRQSAKVSLKAREVLILCSLEERTAQMEHILRSSVVQSRYG
consensus>50 lt#...eL..iL.eLTqL...e.akVaLrAR#VLIa.hlp...y#$rHnq.eS!flSavd
Tni_CD_domain MYGHDFHP...ENLQKLILSETSIFDILHDFFYHTNAAVGNAALEVYVRRAYTSYDITCL
human_CD_domain MYGHQFCI...ENLQKLILSETSIFDVLPNFFYHSNQVVRMAALEVYVRRAYIAYELNSV
ther_CD_domain ETGWSHRRPDREVLKEVVDSKYTVFDVLTLFFAHEDPYVALAALEVYVRRAYRAYNLREV
consensus>50 myGhdf.....EnLqkl!lSets!FD!L.dFFyH.#..V.mAALEVYVRRAY.aY#l..v
Tni_CD_domain QHLALSGELGVVHFQFILPTGHPNRIPISQSEIELASAQDQEGIPAELCTAAMRKCHHRT
human_CD_domain QHRQLKDNTCVVEFQFMLPTSHCQRM..................................
ther_CD_domain RYHD.EERPYFIDWDFV.PSSPATPVE.................................
consensus>50 qh.ql.de..v!ef#FilPtsh.nri..................................
Tni_CD_domain GALAAFESFDQFVQYADELLLVHDFASSATVRREDLAALQEGSESRDSTSINVGSDFKP
human_CD_domain GGMVSFRTFEDFVRIFDEVM.......................................
ther_CD_domain NDFKRIHSISDMTYLARRTRL......................EPIRKGVIVPCKDLLDA
consensus>50 g.l..f.sfd#fv..ade.ld.........................r....i........
Tni_CD_domain IDADNEALLEPIHILMIGVRDSGESDDSAVSRRFGNFCRAHRHELHQKRIRRITFMLLIK
human_CD_domain .....GCFEPIHILNVAIKTDCDIEDDRLAAMFREFTQQNKATLVDHGIRRLTFLVA..
ther_CD_domain EEALSRALEVLPAVVNVAVRDAEGKNDEEILALIKPWVQNSKADLLARRVRRLTFICGRN
consensus>50 .da...al.epih!ln!a!rd..e.#Dd.v.amf.nf.qq.kaeL.q.r!RRlTFm....

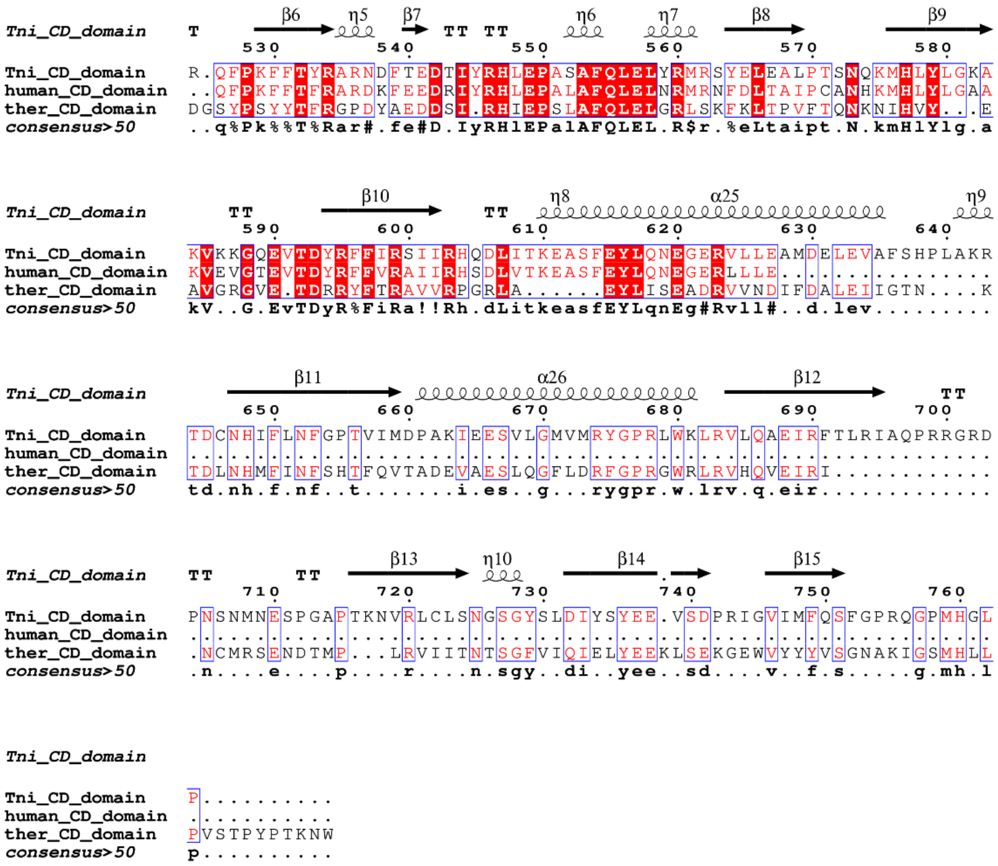

Fig. S6. Sequence alignment of the ACC CD domain. Sequence alignment of the ACC CD domain was performed for *T. ni* (predicted structure by AlphaFold), human (*H. sapiens*, 6G2D) and ther (*T. thermophila*, 5I6H).

Table S1. Cryo-EM data collection, refinement, and validation statistics.

	T. ni ACC (EMD-45956) (PDB 9CV6)
**Data collection and processing**	
Magnification	130,000
Voltage (kV)	300
Electron exposure (e–/Å^2)	53.7
Defocus range (μm)	-1.0 ~ -2.0
Pixel size (Å)	0.664
Symmetry imposed	C2
Initial particle images (no.)	513,450
Final particle images (no.)	154,035
Map resolution (Å)	3.12
FSC threshold	0.143
Map resolution range (Å)	2.9-4.9
**Refinement**	
Initial model used (PDB code)	N/A
Model resolution (Å)	3.10
FSC threshold	0.5
Map sharpening *B* factor (Å^2)	-100
Model composition	
Non-hydrogen atoms	12030
Protein residues	1520
B factors (Å^2)	
Protein	47.91
R.m.s. deviations	
Bond lengths (Å)	0.004
Bond angles (°)	0.854
**Validation**	
MolProbity score	1.23
Clashscore	3.62
Poor rotamers (%)	0.00
Ramachandran plot	
Favored (%)	97.62
Allowed (%)	2.38
Disallowed (%)	0.00

Tables S2. Representative structures of acetyl-coA carboxylase from various species.

Classification	Species	Biotin carboxylase	Central	Biotin carboxyl carrier protein	Carboxyltransferase	Holoenzyme	ACC filament
Eukaryotic	*Saccharomyces cerevisiae*	1W93;1W96	3PGQ; 3K8X; 5I6E	5CSA	1UYS; 1UYT; 1UYV; 1UYR;1OD4;1OD2	5CSL	
	Thermochaetoides thermophila		5I6H; 5I6E; 5I87		5I6H; 5I6F; 5I6G	5I6I (dBCCP-variant)	
	Homo sapiens	3JRX; 3GID; 3GLK	5I87				6G2D; 6G2H; 6G2I
Prokaryotic	*Escherichia coli*	1BNC	N/A	1BDO	2F9Y		
	Staphylococcus aureus				2F9I		
	Mycobacterium tuberculosis				4FB8;4G2R		

N/A indicates domains that are not expected to be present in the ACC.
